# Public Hospital Quality Assessment. Evidence from Greek Health Setting Using SERVQUAL Model

**DOI:** 10.3390/ijerph18073418

**Published:** 2021-03-25

**Authors:** Aspasia Goula, Maria-Aggeliki Stamouli, Maria Alexandridou, Lemonia Vorreakou, Aristeidis Galanakis, Georgios Theodorou, Emmanouil Stauropoulos, Martha Kelesi, Evridiki Kaba

**Affiliations:** 1 Master of Health and Social Care Management, Department of Business Administration, School of Administrative, Economics and Social Sciences. University of West Attica, 12243 Athens, Greece; mstamouli@yahoo.com (M.-A.S.); m.alexadridou@gmail.com (M.A.); lvorreak@gmail.com (L.V.); aristeidhsgal@hotmail.com (A.G.); theodorougeorgios@gmail.com (G.T.); manosfed1326@hotmail.gr (E.S.); 2 Department of Nursing, School of Health and Care Sciences, University of West Attica, 12243 Athens, Greece; mkel@uniwa.gr (M.K.); ekaba@uniwa.gr (E.K.)

**Keywords:** hospital quality, public hospitals, SERVQUAL model, patient expectations, patient perceptions

## Abstract

(1) Background: Health care service quality has been equated with preparedness to provide, accessibility, suitability, adequacy, friendliness and ongoing support and has been connected to service excellence. The main aim of this study was to investigate patients’ perceptions and expectations regarding the quality of health services. (2) Materials and Methods: A cross-sectional analysis was carried out in 5 public general hospitals and convenience sampling was used as the sampling technique. Questionnaires were distributed to inpatients and outpatients and 700 valid questionnaires were returned. The SERVQUAL questionnaire was used for data collection in this survey. (3) Results: Overall, in this study, it became apparent that patients’ expectations as regarding the quality of the provided services were not met. All of the five quality dimensions had a negative gap between patients’ expectations and perceptions. (4) Conclusions: The findings suggested that hospital managers and health care professionals should be interested about patient expectations and subsequently they should search out ways and means to meet them. Open communication with patients, individualized attention, as well as responsiveness to their requirements, polite behavior, trustful atmosphere across the hospital and better physical facilities are the key elements that determine the patient’s judgment about quality.

## 1. Introduction

The quality of services and its management in the modern public health administration has been the priority of reforms in many developed countries over the last few decades. Many measures have been partially or fully implemented in the Greek public health care system, including hospital mergers, decreasing the number of beds in clinics and specialist units, and changing the hospital payment system. The content and reform process was essentially technocratic/managerial in nature, with little respect for the health system’s overall functioning and the needs of the patients [1].

Greece, over the 2008–2018 period, was under an economic crisis. As a result, the health system was underfunded. The public expenditure on health did not exceed 5% of the GDP, a percentage that was significantly lower than that of other developed countries [2]. The economic crisis had made more apparent the need for drastic reforms of the Greek health care system, so that it could equitably and universally provide high-quality services. Unfortunately, after budget reductions were made, the shares of government spending by health care function remained largely unchanged. The Greek health care system is strongly centred in hospitals and the primary care system has not been developed fully [1]. As a result, the inpatient care in 2018 was in the top area of health expenditure (44%) which is the highest proportion in the EU27 (30%) [3,4]. However, Greece is among the OECD countries with the lowest overall response rate for both inpatient and outpatient services [5,6].

Eurobarometer studies suggest a high degree of patient dissatisfaction with Greece’s quality of health care. In the 2014 survey, only 26 percent of respondents in Greece assessed the country’s quality of hospital care as good, while 78 percent thought that the health of hospitalized patients may have ”deteriorated” [7,8].

### Health Services Quality

The term quality is easy to pronounce but it is very difficult to define precisely [9]. The American Society for Quality Control defines quality as “the totality of features and characteristics of a product (or service) that bears on its ability to satisfy stated or implied needs” [10]. 

Service quality has been equated with preparedness to provide, accessibility, suitability, adequacy, friendliness and ongoing support [10] and has been connected to service excellence, differentiation, competitive characteristics, and is extremely important for customer loyalty and retention [11,12,13,14]. 

Compared to the quality of products, the quality of services is more difficult for customers to determine, as both the results obtained (technical quality) and the service delivery process (functional quality) are evaluated [15]. According to Parasuraman, Berry and Zeithaml [16]: “unlike good quality, service quality is an abstract and elusive construct because of three features unique to services: intangibility, heterogeneity and inseparability of production and consumption”. Therefore, an effective approach to assess quality is to evaluate the perceptions of customers about quality. Kano et al. [17] were the first to define product/service characteristics by taking into account their ability to establish customer satisfaction.

Concerning health service quality, the main difference between health services and other services is that they are based on patients’ needs and not on customers’ desires [18]. As a result, assessing quality in the health-care industry is becoming more complex. In addition, it is provided in a unique manner. Professionals offer health-care services, but there is frequently no visible result. In addition, as Taner and Antony stated, patients are quite unique as “customers” due to their low expertise and asymmetry of knowledge in comparison with health care professionals [6]. Patients are not skilled and do not have the needed knowledge to diagnose and treat [19].

The Institute of Medicine included patient satisfaction as an important element of health care outcomes in defining the dimensions of quality. In particular, it mentioned that “quality of care is the degree to which health services for individuals and populations increase the likelihood of desired health outcomes and are consistent with current professional knowledge” [20]. 

Since 1900, the notion of patient satisfaction has been an area of scientific study. The key emphasis at the beginning of modern medical science was to cure the patient or alleviate his or her suffering [21]. However, a new idea was introduced to the scientific community at the beginning of the 20th century that involved the assessment not only of the outcome of the illness, but also of the treatment provided [22,23].

Donadebian introduced a new dimension to the definition of patient satisfaction by connecting patient satisfaction to the quality of the health services offered, and argued that the measurement of service quality should provide an analysis of the system to achieve a given level of quality of health care (the characteristics of physicians, hospitals and employees); of the process (interaction with the structure) and of the outcome (what happens to the patient after the medical act) [24]. 

Studies have revealed that a patient’s satisfaction is affected by the therapeutic relationship between the doctor and the patient, the therapeutic efficacy and the patient’s health-related quality of life [25]; the doctor’s technical skills and the quality of information given to the patient, the hospital environment, the quality of infrastructure and support services, patient’s previous experiences and the cost of services as well as the fulfillment of his needs and his expectations [26,27,28,29,30]. 

Particularly, understanding patients’ expectations and perceptions of the provided services is a key element of the assessment of quality [31] and can be measured by comparing the above two dimensions [32,33]. If the services provided are more than their expectations, those services are considered excellent [33]. A difference between the two does not necessarily indicate low-quality service, but rather that the patient requirements have not been met, which lead to his/her dissatisfaction [34]. 

The main aim of this study was to investigate patients’ perceptions and expectations regarding the quality of health services offered by the 5 public hospitals under study. Additional objectives were to evaluate the gap of each dimension, to determine whether the sociodemographic factors influence it and to highlight which dimension is the most important for patients.

## 2. Materials and Methods

### 2.1. The SERVQUAL Quality Questionnaire

The SERVQUAL—The Service Quality Questionnaire [35] is a methodology and at the same time a tool for analysis, development and measurement of service quality at the functional rather than technical level [36]. Its creators emphasize that there are several factors that are commonly important to all services and, most significantly, are crucial to determining quality [34]. Parasuraman, Berry and Zeithaml defined the quality of service as the difference between the expected service and the perceived service. 

Initially, they had proposed 10 dimensions of services quality. Subsequently, they concluded at 5 dimensions that included 22-items. The dimensions are defined as follows: [35,37]

“Tangibles: physical facilities, equipment and appearance of personnel. Reliability: ability to perform the service accurately and dependably. Responsiveness: willingness to help customers and provide prompt service. Assurance: employees’ knowledge, courtesy and ability to convey trust and confidence. Empathy: caring and individualized attention provided to customers”.

Despite the fact that the method has received negative reviews from various academics [6,34], SERVQUAL measurement is the most popular scale used to evaluate service quality [38] including hospitals worldwide [39] and in Greece too [6,34,40].

### 2.2. Participants and Procedure

The survey was carried out in 5 public general hospitals in the Region of Attica, Greece. The selection criterion for these hospitals was the large number of patients they frequently treated and accommodated. According to the Greek Ministry of Health [41], the Attica region has 23 General Public Hospitals that provided health services to 634,691 patients in 2019. In the same year, the five hospitals we studied provided health care services to 172,968 patients (27 percent of the whole access population). Secondary and tertiary care, as well as advanced primary care, are all offered by the specific hospitals.

The research design was a cross-sectional analysis and the sampling technique used was convenience sampling. This is a non-probability sampling method where the sample is taken from a group of patients easy to contact or reach (i.e., those who were more willing to participate in the survey). The 700 valid filled-in questionnaires corresponded to a response rate of 70%. 

The participants of the research were adults (over 18 years), patients who understood and spoke fluently the Greek language and patients not hospitalized with covid-19. The research was conducted from the 7 November 2020 to the 31 December 2020.

All participants were provided with a written consent form, by means of a declaration, as a separate part of the questionnaire, before proceeding with the completion of the survey. Data collection guaranteed anonymity and confidentiality. All subjects were informed of their right to refuse or discontinue participation in the study, according to the ethical standards of the Helsinki Declaration.

### 2.3. Research Instrument

The research method used in this study for data collection was the Greek version of the SERVQUAL Quality Questionnaire which had been validated for Greek health settings by Christoglou, Vassiliadis and Sigalas [40]. It consists of 22 pairs of questions [expectations (E) and perceptions (P)] that make up the five quality dimensions. The dimensions are the following: (i) Tangibles (4 items); (ii) Reliability (5 items); (iii) Responsiveness (4 items); (iv) Assurance (4 items); (v) Empathy (5 items). All the questions were ranked on a 7-point Likert scale, ranging from 1-totally disagree to 7-totally agree, which means that higher scores show higher expectations and better evaluation of the received services. The questionnaire had two more sections, both of which were parts of the original research tool: one regarding the demographic characteristics of the respondents (5 items) and another one where the patients were asked to allocate a total of 100 points among the five quality dimensions considering how important each dimension was to them.

### 2.4. Statistical Analysis

Data analysis was carried out with SPSS 26 (IBM, Athens, Greece). The five quality dimensions were calculated as mean values of the variables/questions that composed each one of them. This was done for both expectations and perceptions and thus ten new variables (five pairs of perceptions-expectations for each service quality dimension) were created. Afterwards, the gap between perceptions and expectations was calculated for these variables by subtracting expectations from perceptions [P-E]. Kolmogorov–Smirnov and Shapiro–Wilk tests were applied to assess normality of their distributions. These tests showed a statistically significant deviation from normality. Additionally, their graphical illustration using boxplots displayed many outliers for all the gaps, thus all the statistical tests that were used were non-parametric (Table 1).

Specifically, the non-parametric Mann–Whitney U test was used to determine possible statistically significant differences of the gaps between two independent groups and Kruskal–Wallis H test was used to determine whether statistically significant differences existed between more than two groups (with post-hoc analysis based on the non-parametric Dunn’s test with Bonferroni correction). Additionally, Wilcoxon matched-pairs signed-ranks test was used to check for statistically significant differences between perceptions and expectations for each of the five pairs of the quality dimensions and the non-parametric Spearman’s Rho correlation coefficient was used in order to evaluate for possible correlations between the gaps of perceptions-expectations of the SERVQUAL dimensions.

Regarding the reliability of the questionnaire, Cronbach’s alpha coefficient was calculated separately for each section of the questionnaire which composed the quality subscales, for both expectations and perceptions. Its values ranged between 0.68 and 0.88, [Tangibles: (E) = 0.76, (P) = 0.80, Reliability: (E) = 0.86, (P) = 0.88, Responsiveness: (E) = 0.68, (P) = 0.85, Assurance: (E) = 0.87, (P) = 0.80) and Empathy: (E) = 0.83, (P) = 0.88] and they are considered to be good to excellent. This result was a proof of the questionnaire’s internal consistency. The level of statistical significance was set to α = 0.05.

## 3. Results

### 3.1. Descriptive Analysis of the Sample

The SERVQUAL questionnaire was distributed to 1000 patients of 5 Greek Public Hospitals in the region of Attica and 700 valid questionnaires were returned. A summary of the demographic characteristics of the participants is shown in Table 2. Specifically, 59.3% of the respondents received clinical hospitalization (ClHos) and 40.7% of them were treated in outpatient clinics (OutCl). From 700 respondents, 50.6% were males and 49.4% were females. Regarding the age distribution, most of the respondents (29.4%) belonged to the age group of (50–64), 26.1% belonged to the age group of (35–49), 22.4% to the age group of (65 and over) and the remaining 22.0% to the age group of (18–34). In terms of education level, the majority of the participants (36.6%) were university graduates (UG) with or without a postgraduate degree, 33.6% and 14.5% of them reported that they were secondary (SE) and postsecondary education (PSE) graduates respectively, while 15.3% had completed compulsory education (CE). Finally, regarding their marital status, the majority (47.3%) of the participants reported that they were married (Table 2).

### 3.2. Gap Analysis

The gaps for the SERVQUAL dimensions were calculated by subtracting expectations from perceptions [P-E] and their values would specify the overall quality of the health services as assessed by the participants. Further, a mean quality gap was calculated for each dimension as a mean value of the gaps of the questions/items that composed each one of them. A negative value of the gap indicated that the quality of the provided services was lower than expected, while a positive gap value indicated higher-quality services than expected. 

The following table (Table 3) shows that the gaps for all the dimensions of quality had a negative value, which means that the provided health services were lower than expected for all the dimensions. The Tangibles dimension was the one with the highest gap score (−0.92), followed by Assurance (−0.81) and by Empathy (−0.72), while Responsiveness was the dimension with the lowest gap score (−0.62) followed by Reliability (−0.70), which means that the last two were the dimensions where the difference between perceptions and expectations was the smallest. It should be noted at this point that the overall mean gap was negative and of particular interest is the finding that none of the items had a positive gap. 

In regard to the patients’ expectations, Table 3 shows that Assurance was the dimension with the highest score (6.73) followed by the dimensions of Reliability (6.69), Tangibles (6.67), Empathy (6.56) and Responsiveness (6.51). While, in terms of patients’ perceptions, in Table 3 it is shown that the dimension with the highest value was Reliability (5.99), followed by Assurance (5.92), Responsiveness (5.89), Empathy (5.84) and Tangibles (5.75).

Τhe five statements with the highest and the lowest scores on expectations are presented in Table 4. These are the services for which patients expect the highest and the lowest level of quality respectively. It can be seen that four out of five statements with the highest scores concern the dimensions of reliability and assurance and one concerns the tangibles dimension. This finding indicated that patients place more importance on the reliability and assurance of health services than on tangibles, responsiveness and empathy. Nevertheless, they considered the hygiene of the instruments of high importance. The above findings are also confirmed by the fact that three out of five statements with the lowest scores were concerned the empathy dimension, and the other two concerned tangibles and responsiveness. However, it is worth mentioning that among the five statements with the lowest scores on perceptions, the item with the highest score belonged to the responsiveness dimension.

In terms of patients’ perceptions, Table 5 presents the five statements with the highest and the lowest scores respectively. These are the services that patients rated as the best and worst regarding their perceived quality. From Table 5 it is shown that four out of the five statements with the highest scores on perceptions concerned the dimensions of assurance and tangibles and the fifth considered the reliability dimension. In particular, one of the items of assurance and one of the items of tangibles dimensions were also among the five statements with the highest scores in expectations and the same holds for the item of the reliability dimension. These statements were: “The behavior and attitude of hospital staff should inspire confidence in patients”, “The tools they use for my treatment are always neat and clean” and “The hospital keeps its records properly (e.g., Medical Record, Appointments, etc.)” (Table 4 and Table 5). 

Regarding the statements with the lowest scores on perceptions, two out of five concerned the tangibles dimension and the remaining three concern the dimensions of assurance, responsiveness and empathy. It is worth noting that the item of the assurance dimension was also among the five statements with the highest scores in expectations. This item as well as the two items of the tangibles dimension were the only ones with a gap greater than −1.00.

#### 3.2.1. Analysis of the Difference in Perceptions-Expectations

A Wilcoxon matched-pairs signed-ranks test was used to assess whether there were significant differences between expectations and perceptions of the patients for the five service quality dimensions. Tests proved to be significant (*p* < 0.001) for the pairs of perceptions-expectations of all the dimensions. This result indicates that the median perceptions’ scores were statistically significantly lower that the median expectations’ scores for all the dimensions (Table 6).

#### 3.2.2. Gaps Correlations

Statistical analysis with Spearman’s Rho correlation showed statistically significant (*p* < 0.01) strong positive correlations (Table 7) among all the gaps of perceptions-expectations of the SERVQUAL dimensions, apart from the correlations between the gaps of: (i) tangibles-responsiveness and (ii) tangibles-empathy, which are significant, positive and moderate (0.543 and 0.573 respectively). From the same table it was also noticed that the strongest correlations were observed between: (i) the gaps of reliability and responsiveness (0.743), (ii) the gaps of reliability and assurance (0.734) and (iii) the gaps of assurance and empathy dimensions (0.710).

### 3.3. Impact of Sociodemographic Factors on Gap

#### 3.3.1. Type of Service Factor

The non-parametric Mann–Whitney U test was used to evaluate the impact of “Type of Service” on the gaps of perceptions-expectations for the dimensions of SERVQUAL. The test was statistically significant for all the gaps except for the one of tangibles dimension, (UGT = 56,425.50, *p* = 0.300, UGRel = 47,382.50, *p* < 0.001, UGRes = 49,536.00, *p* < 0.001, UGA = 51,448.50, *p* = 0.003, UGE = 49,519.50, *p* < 0.001) (Table 2). Additionally, the test indicated that the gaps of all the dimensions were statistically significantly smaller for the patients who received clinical hospitalization than for those who were treated in outpatient clinics (GRel: MedianOutCl = 0.6 MedianClHos = 0.4, GRes: MedianOutCl = 0.5 MedianClHos = 0.25, GA: MedianOutCl = 0.75 MedianClHos = 0.5, GE: MedianOutCl = 0.6 MedianClHos = 0.4). 

#### 3.3.2. Gender Factor

The gender factor did not seem to affect patients’ perceptions as regarding the quality of health services, since Mann–Whitney U test was not statistically significant for any of the gaps of perceptions-expectations of the SERVQUAL dimensions (UGT = 56,125.00 *p* = 0.055, UGRel= 57,029.00, *p* = 0.113, UGRes = 59,713.00, *p* = 0.565. UGA = 57,659.00, *p* = 0.178, UGE = 57,492.00, *p* = 0.159) (Table 2). 

#### 3.3.3. Age Groups Factor

Regarding the “Age Groups” factor, statistical analysis using the Kruskal–Wallis H test showed statistically significant differences among the age groups for all the gaps of SERVQUAL dimensions apart from the responsiveness one (HGT = 9.94, *p* = 0.02, HGRel = 30.02, *p* < 0.001, HRes = 5.53, *p* = 0.137, HGA = 17.40, *p* = 0.001, HGE = 10.57, *p* = 0.014) (Table 2). The subsequent post hoc analysis for the gaps, based on the Dunn’s test adjusted with Bonferroni correction, identified that statistically significant differences exist for: (i) Tangibles Gap for the pair [(65 and over) vs. (18–34)], [*p* = 0.02, median (65 and over) = 1.0, median (18–34) = 0.5)], (ii) Reliability Gap for the pairs: (a) [(65 and over) vs. (18–34)], [*p* < 0.001, median (65 and over) = 0.2, median (18–34) = 0.8)], (b) [(65 and over) vs. (35–49)], [*p* = 0.003, median (65 and over) = 0.2, median (35–49) =0.6)] and (c) [(50–64) vs. (18–34)], [*p* = 0.002, median (50–64) = 0.4, median (18–34) = 0.8)], (iii) Assurance Gap for the pairs: (a) [(65 and over) vs. (18–34)], [*p* = 0.001, median (65 and over) = 0.5, median (18–34) = 1.0], and (b) [(65 and over) vs. (35–49)], [*p* = 0.01, median (65 and over) = 0.5, median (35–49) = 0.75, and finally (iv) Empathy Gap for the pair [(65 and over) vs. (18–34)], [*p* = 0.01, median (65-over) = 0.2, median (18–34) = 0.8)]. It can be seen that older patients had significant smaller gaps than the younger patients for all the dimensions except for responsiveness, where the test was not statistically significant. 

#### 3.3.4. Marital Status Factor

Based on the analysis with Kruskal–Wallis H, in order to study the impact of “Marital Status” factor on the gaps of perceptions-expectations of SERVQUAL dimensions, the test proved to be statistically significant only for the gaps of reliability and assurance dimensions (HGT = 6.29, *p* = 0.098, HGRel = 18.10, *p* < 0.001, HRes = 6.28, *p* = 0.099, HGA = 13.09, *p* = 0.004, HGE = 6.37, *p* = 0.095) (Table 2).

The subsequent post hoc analysis for the gaps, based on the Dunn’s test adjusted with Bonferroni correction, proved statistically significant differences for: (i) Reliability Gap- or the pairs: (a) [widow vs single], [*p* < 0.029, median (widow) = 0.2, median (single) = 0.8)] and (b) [married vs single], [*p* < 0.002, median (married) = 0.4, median (single) = 0.8)] and (ii) Assurance Gap for the pair [married vs single], [*p* < 0.048, median (married) = 0.5, median (single) = 0.75)]. It can be observed that the gaps of the unmarried patients were larger than those of married patients and widows as regards reliability and the assurance dimension. 

#### 3.3.5. Education Level Factor

The non-parametric Kruskal Wallis-H test, was used to evaluate the impact of “Education Level” on the gaps of SERVQUAL dimensions. The test was statistically significant for all the gaps except for the gap of responsiveness (HGT = 13.43, *p* = 0.004, HGRel = 15.83, *p* = 0.001, HRes = 3.40, *p* = 0.334, HGA = 11.81, *p* = 0.008, HGE = 11.47, *p* = 0.009) (Table 2).

From the post hoc analysis, it was shown that statistically significant differences exist for: (i) Tangibles Gap-for the pairs (a) [CE vs. HE)], [*p* = 0.005, median (CE) = 0.5, median (HE) = 1.0)] and b) [SE vs. HE)], [*p* = 0.04, median (SE) = 0.75, median (HE) = 1.0)], (ii) Reliability Gap-for the pairs: (a) [CE vs. HE)], [*p* = 0.003, median (CE) = 0.2, median (HE) = 0.8)] and (b) [SE vs HE)], [*p* = 0.013, median (SE) = 0.4, median (HE) = 0.8 (iii) Assurance Gap-for the pairs: (a) [CE vs. HE)], [*p* = 0.032, median (CE) = 0.5, median (HE) = 0.75)] and (b) [SE vs. HE)], [*p* = 0.028, median (SE) = 0.5, median (HE) = 0.75 and (iv) Empathy Gap-only for the pair [CE vs. HE)], [*p* = 0.023, median (CE) = 0.2, median (HE) = 0.6)].

#### 3.3.6. Patients’ Prioritization of Quality Dimensions

**Figure 1:**
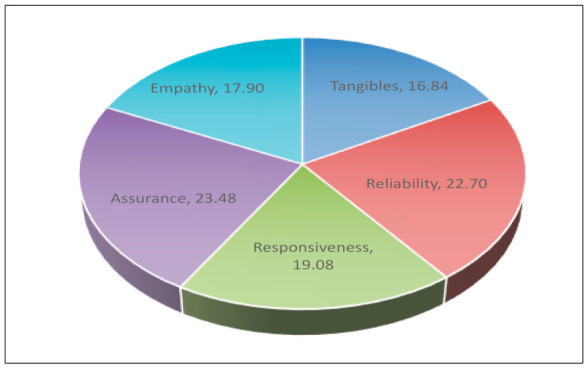
Prioritization of quality dimensions.

As it is mentioned in the third section of the questionnaire, patients were asked to allocate a total of 100 points among the five quality dimensions considering how important each dimension was to them. The allocation of the points had to be done in such a way that the highest score was to be allocated to the most important dimension, the lowest score was to be allocated to the least important dimension, and the sum of all scores had to be equal to 100. According to patients, the five quality dimensions were ranked from the most important to the least as follows: (1) Assurance (23.48), (2) Reliability (22.70), (3) Responsiveness (19.08), (4) Empathy (17.90), and (5) Tangibles (16.84) as it is shown in Table 8 and in Figure 1.

## 4. Discussion

Researching and analyzing the opinion of patients-users is considered as one of the most important indicators for evaluating health services’ provided quality. It is a key administrative element that has been taken into account in most health care system reforms. It is considered as a means of feedback on the health system from society, which in its discretion, strengthens the problem identification and resolution, identifies the most cost-effective management techniques and therefore contributes to the implementation of programs that continuously improve the quality of health services [42,43].

In this study, 700 patients from 5 general hospitals in the region of Attica participated, aiming to assess the hospitals’ quality. The research tool used was the Greek version of SERVQUAL [40]. It consists of 22 pairs of questions (expectations and perceptions) that make up five quality dimensions. 

Overall, by this study, it was made clear that patients’ expectations as regards the quality of the provided services are not met. All of the five quality dimensions have a negative gap between patients’ expectations and perceptions. This finding is similar to most of the studies using the SERVQUAL instrument in Greece [4,34], as well as in other countries [44,45,46,47,48,49,50,51,52]. 

Concerning the Greek public hospitals, a few studies highlighted that one reason for this negative gap is the economic crisis that the country has been undergoing for more than 10 years. Even nowadays, there are still shortages in supplies, facilities and staff with unfavorable consequences on the quality of the provided hospital care. Furthermore, the reduction in the number of employees, the drastic cuts in wages and the abolition of the thirteenth monthly wage, due to the crisis, tend to have increased job dissatisfaction, which can indirectly lead to poor job results, absenteeism and low effort [53,54,55,56]. 

Although the gap scores for SERVQUAL were all negative, it is important not to jump to a negative conclusion about the services offered. In fact, the perception assessment scores that were recorded for hospitals were above the neutral value of “4”, for all the dimensions. These scores were cancelled out by higher expectation scores resulting in negative gap scores [33].

Regarding the quality dimensions of the instrument, the highest gap appeared in the tangibles dimension (−0.92). This result was in line with the studies of Arasli et al. [52] and Ahmad et al. [48], but disagreed with the findings of the studies of Fan et al. [47], and Purcarea et al. [38]. The patients participated in our study seemed to be the least satisfied with the physical environment, the equipment, the hygienic conditions, the hospital cleanliness and the appearance of health employees. According to Karrasavidou et al. [6], tangibles is the easiest quality dimension for patients to evaluate since they do not have the knowledge and the expertise to question the doctors’ decision about their health problems. A comfortable and appealing physical environment, up-to-date and well-maintained equipment, a clean environment and an attractive employee appearance help patients to relax and deal better with their anxieties. If these parameters are absent, it is highly possible for a patient to feel dissatisfied.

Apart from the tangible dimension, there were negative gaps in the other dimensions as well. Specifically, these gaps in descending order were: assurance (−0.81), empathy (−0.72), reliability (−0.70) and responsiveness (−0.62). These dimensions mainly refer to the interpersonal relationships [57], transactions and contacts that patients have with health care professionals. According to Babakus and Mangold [58], negative gaps in these dimensions are a sign of deeper underlying problems in the quality of hospitals. As Papanikolaou and Zygiaris have mentioned, “low scores in the above dimensions may reflect the inability of the service to hire and retain high-quality professionals, evaluate or reward performance or provide adequate training” [34].

These four dimensions reflect the patients-health care employees’ relationship and particularly the doctor-patient relationship. This is a relationship that mainly focuses on physicians’ expertise, experience and ability to help a patient [6]. Most of the time patients act and judge emotionally. They observe if doctors are genuinely concerned for their patients, if they respond to them appropriately and if they are able to assist them [59]. The doctor-patient relationship is an essential element in patient satisfaction [60]. Felleti et al. have shown that this relationship can predict patient satisfaction and in particular explains 24% of variability of it [61].

Concerning these four quality dimensions, the findings of the present study were similar to other studies carried out in in Greece [34]. It seems that health care professionals have little time to provide sympathy and reassurance, to pay individualized attention to patients, to inspire confidence or to make patients to feel safe.

These findings were further supported by analyzing the patients’ responses to the question of which quality dimension they considered as the most important for assessing the overall quality of the provided health services. According to their answers, the most important dimension was assurance followed by reliability, responsiveness and empathy. This classification of dimensions was consistent with the results of other surveys both nationally and internationally [40,44,45]. 

In fact, this finding highlights the impact that health care professionals, and especially doctors, have on patients’ assessment of quality and it is a notification from patients to health care professionals. Patients seek out health care professionals/doctors with a real interest in patients’ problems and a willingness to try to solve them, who are constantly informed, courteous, to promote a sense of trust and security, without complaining about the workload, who manage their time rationally and who understand the specific needs of each patient. Practically, patients want to be at the center of the health care system [6,40]. 

The dimension of tangibles in this study, as in most of the studies, emerged as the least important for assessing the health services’ quality [4,45,62]. 

It’s worth mentioning that the results of the present study indicated that when patients’ expectations in one dimension were not met, they might not be met in other dimensions as well, particularly when the correlations were strong. The same result was reached by Manulik S et al. [63]. Inadequacy in one of the dimensions that was particularly important to the patients would almost certainly lead to lower ratings in the others. This result showed that quality was a holistic and indivisible concept. Although it could be described by a set of dimensions with different aspects of it, in its core, it remained inseparable.

In this point, it is worth mentioning that in our sample the number of in-patients was much higher than the number of outpatients, which is not the usual situation. This was due to the covid-19 pandemic crisis, where fewer outpatients were visiting hospitals on a daily basis as they were concerned about the virus, and also because hospitals’ policies were prohibiting outpatient visits. Regarding gender, the result was similar to other studies and indicated that the perceived quality gap did not differ between female and male participants [34,64].

In relation to age, the results indicated that older patients had a better opinion of the quality of health services than the younger ones and the result was in line with other studies [34,65]. An empirical analysis in 31 countries concluded that the older a patient is, the more pleased they tend to be with a country’s healthcare system [66]. One possible explanation of this result is that older people, due to their old age, pay more visits to the hospitals and therefore they have more previous experience. In future research, it will be important to investigate further this finding and learn how prior perceptions influence the perceived quality of a specific health service. 

Concerning marital status, the unmarried patients considered the quality of services worse compared to married patients and to widow ones. The result was similar to other studies as well [34]. 

Referring to educational level, the patients with a higher educational level had larger gaps than patients with lower educational level, which also means that they had a worse perception on the quality of services for those dimensions. The literature stated that the level of patient education is an important factor in the perception of quality. In particular, people with low level of education have lower expectations and thus state that they are more satisfied with the doctor-patient relationship, the hospital’s facilities and equipment. This may be due to their limited knowledge and inability to judge the services provided. In contrast, highly educated people report lower satisfaction as they expect more [34,67]. 

### Limitations of Study

This study has some limitations. Firstly, the sampling technique used was convenience sampling, which introduced a systematic selection error and did not allow the results to be generalized. Further, the findings of the survey referred to five general hospitals in Attica, so the results can only be generalized to these hospitals and may not reflect the patients’ expectations and perceptions regarding the quality of public health services offered in the region. Future qualitative research that will thoroughly examine the role of personality-related variables or the state of patients’ health and the type of the disease entity in determining health quality expectations would benefit in more accurate results.

## 5. Conclusions

This study made an attempt, by using the SERVQUAL instrument, to evaluate patients’ expectations and perceptions regarding the quality of offered public health services. The findings suggested that hospital managers and health care professionals should be more concerned about patient expectations and should find ways and approaches to fulfill them. Open communication with patients, individualized attention, sensitivity to their requirements, polite behavior, a trustful atmosphere across the hospital and better physical facilities are the key elements that determine the patient’s judgment about quality. The results of our research were similar to those of other surveys [6,34,40] conducted 10 or 15 years ago and essentially before the economic crisis in the country. The human aspect quality gap was found to be the most critical area for development, and consequently an indicator of overall service quality assessment. Consequently, the health care quality could be systematically measured and hospital managers could take into account the results of these measurements, aiming at the development of policies that will upgrade the quality of health care services. Moreover, health policy makers need to reconsider and foster a public dialogue on the health budget, which could be regarded as a developmental tool rather than a financial burden, with an emphasis not only on the economic development but also on the wellbeing of people.

## Figures and Tables

**Table 1:** Normality tests for SERVQUAL dimensions.

Tests of Normality						
	Kolmogorov-Smirnov ^a^			Shapiro-Wilk		
	Statistic	df	Sig.	Statistic	df	Sig.
GT	0.119	700	0.000	0.963	700	0.000
GRel	0.127	700	0.000	0.932	700	0.000
GRes	0.128	700	0.000	0.941	700	0.000
GA	0.125	700	0.000	0.937	700	0.000
GE	0.116	700	0.000	0.937	700	0.000

^a^ Lilliefors Significance Correction. GT: Gap Tangibles, GRel: Gap Reliability, GRes: Gap Responsiveness, GA: Gap Assurance, GE: Gap Empathy.

**Table 2:** Demographic characteristics of the respondents (*n* = 700) and evaluation of the impact of demographic characteristics on SERQUAL dimensions.

		Frequency	Percent	Tangibles	Reliability	Responsiveness	Assurance	Empathy
				Test *p*-Value	Test *p*-Value	Test *p*-Value	Test *p*-Value	Test *p*-Value
**Type of service**	OutCl +	285	40.7	U * = 56,425.50 *p* = 0.300	U * = 47,382.50 *p* < 0.001	U * = 49,536.00 *p* < 0.001	U * = 51,448.50 *p* = 0.003	U * = 49,519.50 *p* < 0.001
	ClHos ++	415	59.3					
**Gender**	Male	354	50.6	U * = 56,125.00 *p* = 0.055	U * = 57,029.00 *p* = 0.113	U * = 59,713.00 *p* = 0.565	U * = 57,659.00 *p* = 0.178	U * = 57,492.00 *p* = 0.159
	Female	346	49.4					
***Age Groups***	*18–34*	*154*	*22.0*	H ** = 9.94 *p* = 0.02	H ** = 30.02 *p* < 0.001	H ** = 5.53 *p* = 0.137	H ** = 17.40 *p* = 0.001	H ** = 10.57 *p* = 0.014
	35–49	183	26.1					
	50–64	206	29.4					
	65 and over	157	22.4					
**Education level**	CE ^	107	15.3	H ** = 13.43 *p* = 0.004	H ** = 15.83 *p* = 0.001	H ** = 3.40 *p* = 0.334	H ** = 11.81 *p* = 0.008	H ** = 11.47 *p* = 0.009
	SE ^^	235	33.6					
	PSE ^^^	102	14.5					
	HE ^^^^	256	36.6					
**Marital status**	Single	205	29.3	H ** = 6.29 *p* = 0.098	H ** = 18.10 *p* < 0.001	H ** = 6.28 *p* = 0.09	H ** = 13.09 *p* = 0.004	H ** = 6.37 *p* = 0.095
	Married	331	47.3					
	Divorced	98	14.0					
	Widow	66	9.4					

+ Outpatient Clinics, ++ Clinical Hospitalization, ^ Compulsory Education, ^^ Secondary Education, ^^^ Postsecondary Education, ^^^^ Higher Education, * Mann–Whitney U test, ** Kruskal–Wallis H test.

**Table 3:** Results for Expectations, demographic characteristics of the respondents by quality Dimension.

	Question/Item	Expectations	Perceptions	Gap
**Tangibles**	Hospital should have up-to-date equipment	6.74	5.51	−1.23
	The facilities of the hospital (e.g., waiting room/hall, clinics, wards, toilets) should be visually appealing	6.49	5.10	−1.39
	The hospital staff should be well dressed and appear neat	6.62	6.14	−0.48
	The equipment used for treatment should always be well maintained	6.82	6.25	−0.57
	**Mean Value**	6.67	5.75	−0.92
**Reliability**	When hospital staff promise to do something by a certain time, they should do it	6.59	5.83	−0.76
	When a patient has a problem, hospital staff should be willing to help him/her	6.68	6.04	−0.64
	Hospital should be reliable and always provide the right services from the beginning	6.77	6.02	−0.75
	Hospital should provide its service at the time it promises to do so	6.65	5.89	−0.76
	Hospital should keep their records accurately (e.g., medical record, appointments, etc.)	6.76	6.19	−0.57
	**Mean Value**	6.69	5.99	−0.70
**Responsiveness**	Hospital staff should keep the patient informed	6.69	6.00	−0.69
	Hospital staff should provide prompt services to patients	6.61	5.77	−0.84
	Hospital staff should always be willing to help patients	6.72	6.09	−0.63
	The staff of the hospital should always respond to patients’ requests, no matter how busy they are	6.00	5.68	−0.32
	**Mean Value**	6.51	5.89	−0.62
**Assurance**	The behavior and attitude of hospital staff should inspire confidence in patients	6.74	6.14	−0.60
	I should feel safe in my dealings with the hospital staff	6.72	6.12	−0.60
	Hospital staff should be consistent and polite to the patients	6.70	6.07	−0.63
	Hospital staff should receive adequate support in order to do their job well	6.75	5.34	−1.41
	**Mean Value**	6.73	5.92	−0.81
**Empathy**	Hospital staff should pay special attention to each patient	6.57	5.94	−0.63
	The operation hours of the hospital should be convenient for all patients	6.40	5.60	−0.80
	Hospital staff should understand and have the knowledge of the health needs of their patients	6.61	5.95	−0.66
	Hospital should have in mind the interests of their patients	6.53	5.76	−0.77
	Hospital staff should understand the specific health needs of their patients	6.67	5.93	−0.74
	**Mean Value**	6.56	5.84	−0.72
**Total Mean Gap**				−0.75

**Table 4:** Statements with the highest and lowest scores on Expectations.

Expectations-Highest Scores	
Item/Statement	Scores
The equipment used for treatment should always be well maintained	6.82
Hospitals should be reliable and always provide the right services from the beginning.	6.77
Hospitals should keep their records accurately (e.g., medical record, appointments, etc.)	6.76
Hospital staff need to receive adequate support in order to do their job well.	6.75
The behavior and attitude of hospital staff should inspire confidence in patients.	6.74
**Expectations-Lowest Scores**	
The staff of the hospital should always respond to patients’ requests, no matter how busy they are.	6.00
The operation hours of the hospitals should be convenient for all patients.	6.40
The facilities of the hospitals (e.g., waiting room/hall, clinics, wards, toilets) should be visually appealing.	6.49
Hospitals should have in mind the interests of their patients.	6.53
Hospital staff should pay special attention to each patient.	6.57

**Table 5:** Statements with the highest and lowest scores on Perceptions.

Perceptions-Highest Scores	
Item/Statement	Scores
The equipment they use for my treatment are always well maintained	6.25
The hospital keeps its records accurately (e.g., medical record, appointments, etc.)	6.19
The hospital staff is well dressed and neat appears.	6.14
The behavior and attitude of the hospital staff inspires confidence in patients.	6.14
I feel safe in my dealings with the hospital staff	6.12
**Perceptions-Lowest Scores**	
The staff of the hospital always responds to the requests of the patients, no matter how busy they are	5.68
The operation hours of the hospital are convenient for all patients.	5.60
Hospital has up-to-date equipment.	5.51
The employees of the hospital receive the appropriate support in order to do their job well	5.34
The facilities of the hospital (e.g., waiting room/hall, clinics, wards, toilets) were visually appealing.	5.10

**Table 6:** Summary of differences between Expectations—Perceptions for the SERVQUAL dimensions (Wilcoxon matched pairs signed ranks test).

	z-Value	*p*-Value	Median Perceptions	Median Expectations
Tangibles: Perceptions-Expectations	−18.098	<0.001	6.00	7.00
Reliability: Perceptions-Expectations	−12.129	<0.001	6.20	7.00
Responsiveness: Perceptions-Expectations	−14.065	<0.001	6.00	6.75
Assurance: Perceptions-Expectations	−17.783	<0.001	6.00	7.00
Empathy: Perceptions-Expectations	−16.167	<0.001	6.00	6.80

**Table 7:** Spearman’s Rho correlation coefficients among the gaps of perceptions-expectations of the SERVQUAL dimensions (*n* = 700).

	GT	GRel	GRes	GA	GE
GT	1.000	0.637 **	0.543 **	0.619 **	0.573 **
GRel		1.000	0.743 **	0.734 **	0.677 **
GRes			1.000	0.693 **	0.690 **
GA				1.000	0.710 **
GE					1.000

** *p* < 0.01.

**Table 8:** Prioritization of quality dimensions.

Dimension	Question	Points
Tangibles	Visually appealing of physical facilities, equipment and hospital’s employees	16.84
Reliability	Ability of the hospital to execute promising services with reliability and accuracy	22.70
Responsiveness	Hospital staff willingness to provide help to patients	19.08
Assurance	Hospital staff with knowledge, good manners and inspiring confidence	23.48
Empathy	Provision of individual interest and attention to patients by hospital’s employees	17.90
**Total**		100

## Data Availability

Restrictions apply to the availability of these data. Data were collected from Post-Graduate Program “Health and Social Care Management” of the Department of Business Administration of the University of West Attica and are available from Goula A. with the permission of MSc Program.
